# Variations in the Structure and Composition of Soil Microbial Communities of Different Forests in the Daxing’anling Mountains, Northeastern China

**DOI:** 10.3390/microorganisms13061298

**Published:** 2025-06-03

**Authors:** Han Qu, Mingyu Wang, Xiangyu Meng, Youjia Zhang, Xin Gao, Yuhe Zhang, Xin Sui, Maihe Li

**Affiliations:** 1 Engineering Research Center of Agricultural Microbiology Technology, Ministry of Education & Heilongjiang Provincial Key Laboratory of Ecological Restoration and Resource Utilization for Cold Region & Key Laboratory of Microbiology, College of Heilongjiang Province & School of Life Sciences, Heilongjiang University, Harbin 150080, China; quhan2241771@163.com (H.Q.); wmy022234@163.com (M.W.); M13555220601@163.com (X.M.); zdzhidian@outlook.com (Y.Z.); agaoxin0218@163.com (X.G.); 13703670569@163.com (Y.Z.); 2 Forest Dynamics, Swiss Federal Institute for Forest, Snow and Landscape Research, CH-8903 Birmensdorf, Switzerland; 3 Key Laboratory of Geographical Processes and Ecological Security in Changbai Mountains, Ministry of Education, School of Geographical Sciences, Northeast Normal University, Changchun 130024, China; 4 School of Life Science, Hebei University, Baoding 071002, China

**Keywords:** cold temperate climate, forest types, original purest forest, microbial structure, soil microbial diversity

## Abstract

Soil microorganisms are crucial in global biogeochemical cycles, impacting ecosystems’ energy flows and material cycling. This study, via high-throughput sequencing in four forests—the original *Larix gmelinii* (Rupr.) Kuzen. forest (LG), the conifer–broad-leaved mixed *Pinus sylvestris* var. *mongolica* Litv. forest (PS), the original pure *Betula platyphylla* Sukaczev forest (BP), and the original pure *Populus* L. forest (PL) in Shuanghe National Nature Reserve, Daxing’anling mountains—explored soil microbial community structures and diversities. The results indicated that the BP and PL forests had the lowest soil bacterial ACE and Chao1 indices, and the original pure birch forest’s Shannon index was higher than that of the poplar forest. The soil’s fungal Chao1 index of the birch forest was higher than that of the larch forests. *Bradyrhizobium* and *Roseiarcus* were the dominant soil bacterial genera; the dominant soil fungal genera were *Podila*, *Russula*, and *Sebacina*. RDA and mantel analyses indicated that soil microbial community structures varied across forest types mainly because of the effective phosphorous and pH levels, soil’s total nitrogen level, and available phosphorus level. This study offers a scientific foundation for cold-temperate-forest ecosystem management regarding soil microbial diversity and community structural changes in different forest types.

## 1. Introduction

Forest ecosystems play a key role in regulating Earth’s regional environments [1]. A variety of interrelated factors, including physical, chemical, and biological components, combine to influence the structure and function of forest ecosystems [2]. They not only provide timber for daily life and promote the development of the forest economy but also effectively regulate the climate, stabilize soil and water structures, purify the atmosphere, and maintain the stability of the biodiversity. The synergistic effect of plant communities and soil microorganisms is the core mechanism of the forest ecosystem’s functional maintenance. The interaction between plant communities and soil microorganisms has profound impacts on material circulation energy flows and ecological stability. Aboveground vegetation of different forest types drives the formation of unique compositional and functional characteristics of underground microbial communities through litter input, root matter, and mycorrhizal symbiotic networks. Significant differences in litter’s chemical compositions and root metabolites between coniferous and broad-leaved forests shape diverse microbial networks [3,4]. This coupling effect not only reflects the ability of vegetation types to screen and regulate microbial communities but also reveals the pivotal role of microorganisms in maintaining ecosystem services. Exploring the interaction between plants and microorganisms in different forest types has important theoretical value and practical significance for analyzing the carbon and nitrogen balance mechanism of forest ecosystems and optimizing ecological restoration strategies.

Soil microorganisms, as an important component of forest ecosystems, can be directly affected by the aboveground vegetation composition and diversity. Different forest types, including different vegetation compositions and diversities, lead to the heterogeneity of the soil environment (e.g., nutrient contents and pH) through differences in the chemical composition of apoplastic materials and the characteristics of root secretion inputs [5], which, in turn, drive the diversity and structure of the microbial community. These microorganisms, while adapting to specific soil physicochemical conditions, will, in turn, react to the soil environment through metabolic activities, forming a feedback mechanism [6]. However, the impacts of changes in the aboveground vegetation on the soil’s microbial composition and diversity may not always be the same. For example, some studies have shown that the soil’s microbial diversity was positively correlated with the aboveground plant diversity [7,8,9]. For example, Zhou’s research on forest ecosystems found that in karst landform areas, different vegetation types can highly explain the changes in fungal Shannon indices but have low explanatory power for bacterial communities [6]. This confirms that fungi are highly dependent on organic matter types, while bacteria are highly dependent on the soil’s total nitrogen differentiation [10,11,12]. However, some studies have not been consistent with those by Zhou [13] and Hu [14]. For example, in Wu et al.’s study (2025) on the Qinghai–Tibet Plateau, the authors found that aboveground biodiversity does not lead to soil microbial diversity, and there is a stronger correlation between the β-diversity of bacterial communities and the vegetation’s carbon and nitrogen inputs [15]. The high diversity level of soil microorganisms may be determined by the stability of the ecosystem and the nutrient content of the soil. Therefore, the current research conclusions on soil microbial changes in different forest types are not consistent. This may be because of the diversity of global forest ecosystem types, different climatic conditions, and even different forest compositions. The results of soil microbial composition and diversity tests are also inconsistent. Therefore, it remains important to explore the composition and diversity of soil microorganisms in different forest types. Therefore, by studying the changes in microbial communities and the main factors driving these changes, we can explore the regulatory mechanisms of different forest types in soil subsurface ecological processes, and thus provide a microbiological perspective for assessing the impacts of human activities on the structure, function, and stability of forest ecosystems [16].

As a typical representative of the compound ecosystem of a cold-temperate-zone coniferous forest and wetland in China, Shuanghe National Nature Reserve has preserved the following typical forest types: cold temperate coniferous forests (*L. gmelinii* and *P. sylvestris* var. *mongolica* Litv.), broad-leaved forests (*B. platyphylla* and *Populus* L.), and mixed coniferous and broad-leaved forests (*Populus* L. and *L. gmelinii*), and there are significant differences in the forest structures and apomictic materials [17]. However, the presence of human disturbances has caused varying degrees of damage to some of the original pure forests in the region. In 1987, a large forest fire in the northern part of the Daxing’anling Mountains did not involve the protected area, but the fire area in the neighboring area amounted to 13.3 thousand square kilometers, which indirectly affected the stability of the microbial community in the protected area [18,19]. This indirectly affected the stability of microbial communities in the reserve.

The metabolic differences of apoplastic materials in different forest types can be directly reflected in the physical and chemical properties of the soil, which, in turn, affect the structure of the soil’s microbial community. We selected undamaged primary forests for sampling, which made the study more accurate. Therefore, the diversity of the forest types in Shuanghe Reserve provides an irreplaceable research scenario for us to investigate the soil microbial community structures in different forest types in the cold temperate zone.

In this study, we selected four iconic tree species in the Shuanghe Nature Reserve and investigated the compositions of the understory soil’s microbial communities, using Illumina high-throughput sequencing, and the relationships between the microbial communities and the soil’s physicochemical properties. We aim to (1) identify differences in the soil’s microbial communities in these four different forest types and (2) elucidate the environmental factors that drive microbial communities in the different forest types. The results of this study provide scientific data for understanding changes in forest ecosystems in the context of global climate change, as well as changes in the soil’s bacterial communities in cold temperate zones. This study also provides a basis for subsequent studies to determine the assembly mechanisms and functions of soil microbes in the understory of cold temperate forests.

## 2. Materials and Methods

### 2.1. Study Area

**Figure 1:**
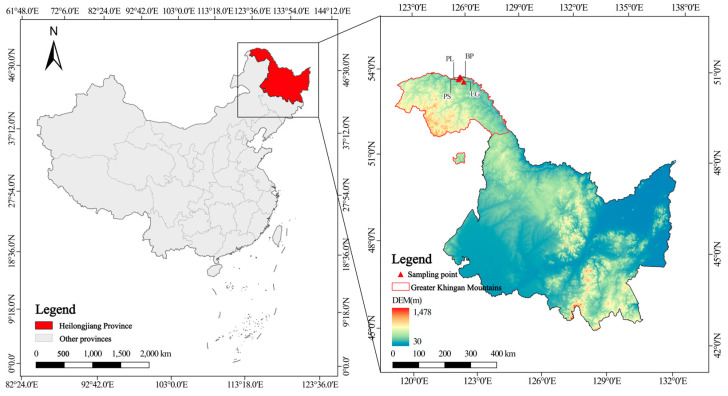
Geographical location of sampling sites in this study.

The study area is located in the northeastern part of the Daxing’anling area, Heilongjiang Province, in Shuanghe Nature Reserve (124°52′48″–125°32′03″ E, 52°54′25″–53°12′08″ N), in Tahe County (Figure 1) and belongs to the cold temperate continental monsoon climate zone, with an average annual temperature of −4.3 °C, an annual precipitation of 460 mm, and a warm and rainy summer, which accounts for 53% of the annual precipitation [17] from many thunderstorms. The frost period is from early September to late May of the following year; the freezing period can be up to seven months long. The average annual snow period is from 165 to 175 days; the average depth of the permafrost is approximately 2.5–3.0 m [20]. In the local low-lying swampy areas, there is an island-like permafrost distribution. The overall topography is not very undulating, with an average elevation of between 200 and 500 m. The climate is relatively humid, with good conditions for plant growth. The vegetation types in this area are mainly divided into two: cold temperate coniferous forest and deciduous broad-leaved forest [21,22]. For each vegetation type, we selected two dominant tree species, namely, the conifer–broad-leaved mixed *Pinus sylvestris* var. *mongolica* Litv. forest (PS) and the original *Larix gmelinii* (Rupr.) Kuzen. forest (LG) (coniferous forest); and the original pure *Populus* L. forest (PL) and the original pure *Betula platyphylla* Sukaczev forest (BP) (deciduous broad-leaved forest), and focused on the composition and diversity of the soil’s microorganisms for sampling and research.

### 2.2. Experimental Design and Soil Sample Collection

Soil samples were collected in August 2024. Before collection, the soil surface of the understory was first cleared of debris, such as large leaf litter and residual plant and animal residues; a soil auger (5 cm in diameter and 20 cm deep) was used to apply a five-point sampling method at a surface depth of approximately 0–20 cm in each plot, and the samples from the five points were mixed together as a single soil sample, with three randomized replicates set up for each treatment, for a total of 12 soil samples. The collected soil samples were mixed, removing stones, roots, etc. Prior to sample collection, the tools were sterilized. The samples were stored in sterile self-sealing bags, preserved using dry ice, and transported to the laboratory. In the laboratory, the samples were divided into two portions: One portion was placed in 15 mL sterilized polypropylene tubes and stored in a cryogenic refrigerator at −80 °C for DNA extraction, while the other portion was air-dried, milled, and sieved through a 2 mm mesh sieve in preparation for the subsequent determination of the chemical properties of the soil.

### 2.3. Measurement of the Soil’s Chemical Properties

The soil’s pH was measured using a pH meter (METTLER TOLEDO, Greifensee, Switzerland) and a 2 mm mesh sieve to remove larger impurity particles and debris from the soil. The resolution was selected, the temperature was selected, and the pH meter was calibrated using standard buffer solutions (e.g., pH 4.01, 7.00, and 10.01) according to the manufacturer’s instructions. The soil samples were mixed with distilled water at a soil-to-water ratio of 1:2.5. The mixture was stirred well and allowed to stand for 30 min. The pH of the supernatant was measured using a calibrated pH meter rinsed with deionized water at the end of the measurement [23,24]. The soil’s total nitrogen content (TN) and soil’s total carbon (TC) were determined using a carbon and nitrogen analyzer (Jena multi EA 4000, Jena, Germany). The soil’s alkaline nitrogen (AN) was determined using the alkaline diffusion method, in which the soil was treated with a sodium hydroxide solution so that the readily hydrolyzable nitrogen was converted to ammoniacal nitrogen by alkaline dissolution, absorbed by a boric acid solution after diffusion, and titrated with a standard solution of hydrochloric acid, and the amount of hydrolyzable nitrogen was calculated [25]. The soil’s effective phosphorus (AP) was determined using sodium bicarbonate leaching and a molybdenum- and antimony-specific cation spectrophotometer, in which the phosphoric acid and ammonium molybdate in the roots formed antimony, phosphorus, and molybdenum mixed heteropolyacids which, at room temperature, can be reduced to molybdenum blue by ascorbic acid. The absorbance value was measured at a wavelength of 650 nm, the content of the effective phosphorus and the absorbance value showed a linear relationship, and the effective phosphorus content in the sample could be calculated using the method of the standard curve [26,27].

### 2.4. DNA Extraction, PCR Amplification, and MiSeq Sequencing

The total DNA of the soil’s microorganisms was extracted from 0.5 g of fresh soil, using a FastDNA® SPIN kit for soil (MP Biomedicals, Santa Ana, CA, USA), and the concentration and quality of the DNA were determined using NanoDrop 2000 (Thermo Scientific, Waltham, MA, USA). The DNA was stored at −20 °C for future reference. The V3-V4 region of the bacterial 16S rRNA gene was amplified using primers 338F (5′-ACT CCT ACG GGA GGC AGCA3′) and 806R (5′-GGA CTA CHV GGG TWT CTA AT3′) (Mori et al., 2014), and ITS1 (5′-CTTGGTCATTTAGAGGAAGTAA-3′) and ITS2 (5′-GCTGCGTTCATCGATGC-3′) were used to amplify the ITS1 region of the fungal ITS rRNA. The polymerase chain reaction (PCR) products were quantified using a QuantiFluorTM-ST blue fluorescence quantification system (Promega, Madison, WI, USA). High-throughput sequencing of the bacterial and fungal communities was performed using Illumina technology, and the quality of the sequences was controlled and filtered using QIIME (1.9.1). The obtained optimized sequences and UPARSE software https://drive5.com/uparse/ were used to remove chimeras, and the non-repetitive sequences were subjected to OTU (operational-taxonomic-unit) clustering analysis according to 97% similarity. The representative sequences of OTUs were annotated for bacterial and fungal classifications using the RDP classifier’s Bayesian algorithm. Finally, the results were compared with the Silva database (Release 138) to determine the specific taxonomic information of the bacterial and fungal OTUs [28].

### 2.5. Bioinformatics and Statistical Analyses

Raw fastq reads for bacteria and fungi were filtered and evaluated using QIIME 2 [29]. The accuracy of the analysis was improved by excluding low-quality sequences < 200 bp in length and sequences with an average quality score of <20. The trimmed sequences were then checked for chimeras, and sequences with chimeras were removed using the Uchime algorithm [30]. Sequences with overlapping sequence combinations of >10 bp were discarded. And the sequences with 97% similarity clustering were combined into operational taxonomic units (OTUs). During clustering, an initial preprocessing step involved the deduplication and quality control of all the sequencing reads, followed by the classification of these sequences into different OTUs according to similarity thresholds. Subsequently, the OTU sequences were extracted from the high-throughput sequencing data [31]. During the analysis, OTUs for bacteria and fungi of specific classifications were obtained using the SILVA database (version 138) [28] and the FUNGuild database [32]. The overall dataset for all the samples was simplified to obtain the minimum number of sequences, which were then used for community analysis [33]. Differences in the soil’s chemical properties among the four sample sites were determined using one-way analysis of variance (ANOVA) and Duncan’s test in SPSS (version 26). Alpha diversity indices, including the ACE index, Chao1 index, Shannon index, and Simpson index, were calculated using the “vegan” package (version 2.6.6.1) in R (version 4.4.1). In addition, the principal coordinate analysis (PCoA) of the microbial beta diversity, based on the Euclidean distance algorithm, was performed using the “vegan” package [34]. Differences in the bacterial and fungal community structures among the soil samples were examined using a ranked multivariate analysis of variance (PERMANOVA), and differences in species abundances across multiple groups were analyzed using the Kruskal–Wallis test [35]. Redundancy analysis (RDA) was performed using the Euclidean distance metric from the “microeco” software package https://academic.oup.com/femsec/article/97/2/fiaa255/6041020?login=false [36]. Mantel tests were performed using the “microeco” package (version 1.8.0) in R software (version 4.4.1) to assess whether the relationship between the soil microbial community’s composition and α-diversity was related to the soil’s physicochemical properties. We chose the soil microbes’ Shannon and richness indices to represent “diversity” and “richness”, respectively, and the microbial Bray–Curtis similarity to represent “composition”. All the data in this study were analyzed using SPSS 26.0 (SPSS, Inc., Chicago, IL, USA) and R (version 4.4.1, Teem 2024). Graphs were generated and analyzed using GraphPad Prism 7.0 (GraphPad Software, Inc., San Diego, CA, USA) and R (version 4.4.1, Teem 2024). A *p*-value of <0.05 was considered as statistically significant.

## 3. Results

### 3.1. Soil’s Chemical Characteristics

All the soil’s chemical properties (TC, TN, AN, AP, and pH) differed significantly among the different forest types (Table 1; *p* < 0.05). The concentrations of TC in the soils of the LG and BP were significantly lower than those in the soils of the PL and PS, and the pH values in the soil of the PS were lower than those in the soils of the other three forests. The concentrations of TN and AN in the soils of the PS were significantly higher than those in soils of the other three forests (Table 1, *p* < 0.05).

### 3.2. Microbial Diversity in Soils of Different Forest Types

In the alpha diversity analysis, the Shannon index was used to measure species diversity, combining species richness and evenness; the Chao1 index and the ACE index focused on estimating species richness, especially for the detection of rare species and the estimation of the number of unobserved species, and the Simpson index mainly reflected the degrees of species dominance and diversity in the community, focusing on the effect of the dominant species on the diversity. Each of these indices focuses on different aspects, and together, they provide comprehensive quantitative information on community species diversity.

**Figure 2:**
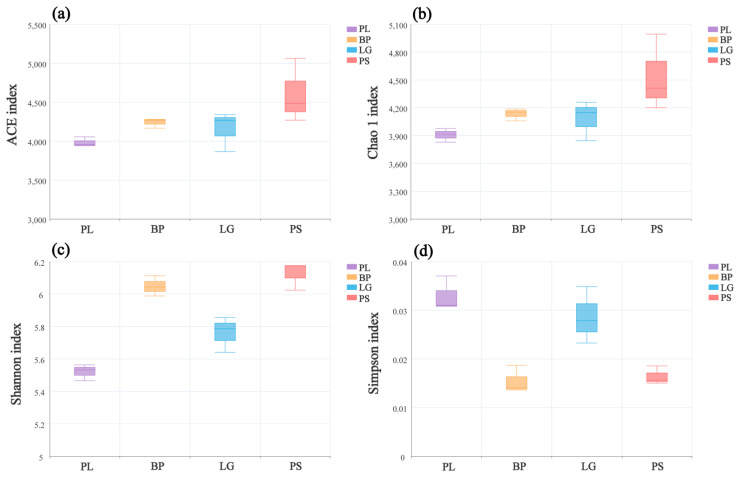
Diversity indices of the soil’s bacterial communities at different sites: ACE (**a**), Chao1 (**b**), Shannon (**c**), and Simpson (**d**). PL: primary pure poplar forest; BP: primary pure birch forest; LG: primary pure larch forest; PS: primary pure camphor pine forest.

Figure 2 shows the changes in the α-diversity indices of the soil’s bacterial communities in the different forests. The ACE, Shannon, and Simpson indices of the soil’s bacterial communities differed significantly, while the Chao1 index did not change significantly in the four different forests. The PL forests had the lowest ACE, Chao1, and Shannon indices but the highest Simpson indices (Figure 2, *p* < 0.05). 

**Figure 3:**
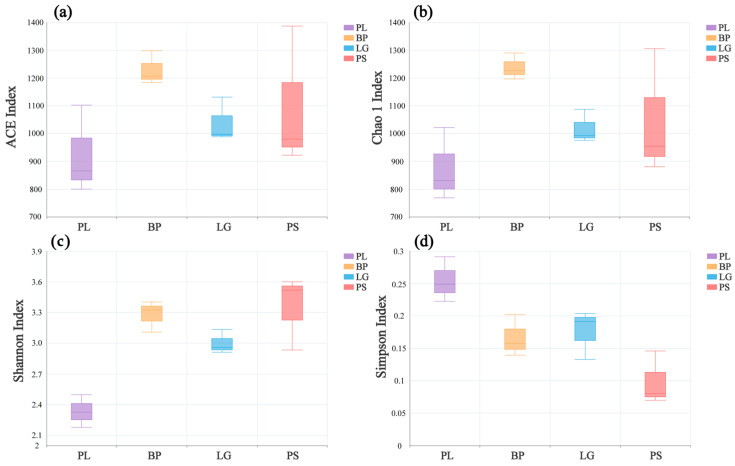
Diversity indices of the soil’s fungal communities at different sites: ACE (**a**), Chao1 (**b**), Shannon (**c**), and Simpson (**d**). PL: primary pure poplar forest; BP: primary pure birch forest; LG: primary pure larch forest; PS: primary pure camphor pine forest.

Figure 3 shows the changes in the α-diversity indices of the soil’s fungal communities in the different forests. There were significant differences among the four indices in the different forest types. As with the soil’s bacterial communities, the PL forests had the lowest ACE, Chao1, and Shannon indices but the highest Simpson indices (Figure 3, *p* < 0.05).

**Figure 4:**
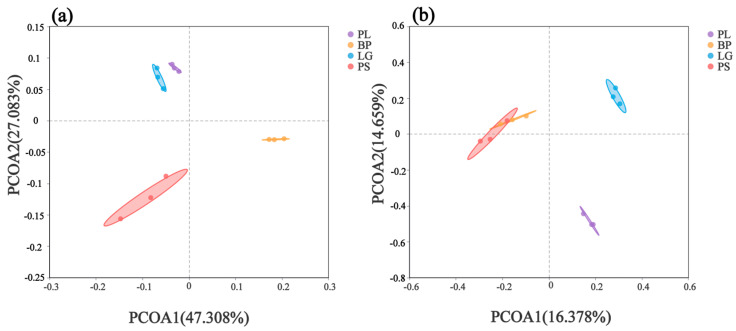
Principal component analysis (PCoA) of the soil’s bacterial (**a**) and fungal (**b**) communities under different treatments. Different colored dots represent different treatments. PL: primary pure poplar forest; BP: primary pure birch forest; LG: primary pure larch forest; PS: primary pure camphor pine forest.

Principal coordinate analysis (PCoA) revealed the β-diversities of the soil’s bacterial and fungal communities in the four different forest types (Figure 4). PCoA showed that the structural compositions of the soil’s bacterial and fungal communities in the different forest types differed significantly (Figure 4, permanova test, *p* < 0.01, Figure S2). The first principal component axis (PCoA1) and second principal component axis (PCoA2) explained the 47.308% and 27.083% variability contribution rates, respectively (Figure 4a). Moreover, the PCoA of the soil’s bacteria (Figure 4a) showed that the soil’s bacterial community compositions in the LG and PL forests were more similar and that the soil’s bacterial compositions in the BP and PS forests were more similar.

The principal coordinates of the soil’s fungal community showed that the first axis (PCoA1) explained 16.378% of the change, and the second axis (PCoA2) contributed an additional 14.659% (Figure 4b). The PCoA plot of the soil’s fungi (Figure 4b) showed that the fungal community’s composition showed higher similarity between the BP and PS, while the LG and PL had more distinct community structures.

### 3.3. Soil’s Microbial Community Compositions in Different Forest Types

**Figure 5:**
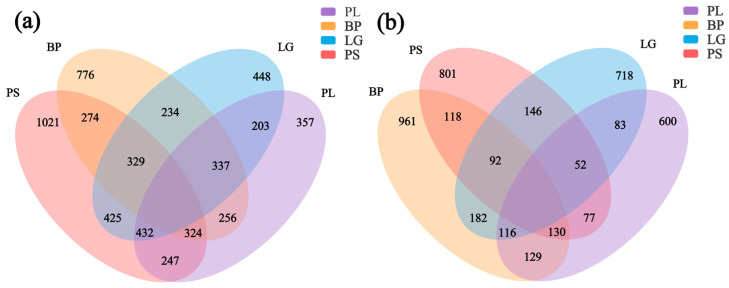
Differential distributions of the soil’s microbial communities under different treatments. Different colors represent different treatment conditions. Overlapping areas represent common bacterial and fungal genera under different treatment conditions. Non-overlapping areas represent bacterial and fungal genera specific to that treatment condition. Numbers represent the numbers of OTUs for bacteria (**a**) and fungi (**b**) under different treatment conditions. PL: primary pure poplar forest; BP: primary pure birch forest; LG: primary pure larch forest; PS: primary pure pine forest.

The soil’s bacterial and fungal community structures were analyzed for the four different forest types. The Venn diagram of the bacterial community included both unique and shared OTUs (Figure 5a). The highest number of unique OTUs was found in the soil in the PS forest (1021), followed by BP (776), LG (448), and PL (357), and the number of bacterial OTUs shared by the four forest types was 247. The Venn diagram of the soil’s fungal communities is shown in Figure 5b. BP had the highest number of unique OTUs (961), followed by PS (801), LG (718), and PL (600), and the number of fungal OTUs common to the four forest types was 129.

**Figure 6:**
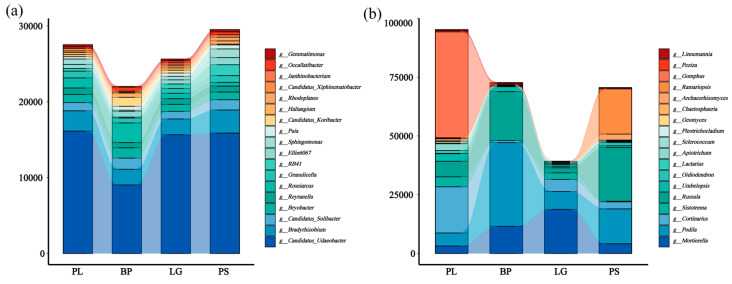
Stacked plots representing the top 18 soil bacterial (**a**) and fungal (**b**) genera in terms of abundances under different treatments. PL: primary pure poplar forest; BP: primary pure birch forest; LG: primary pure larch forest; PS: primary pure pitch pine forest.

The relative abundances of the top three phyla were Verrucomicrobiota (13.1) > Proteobacteria (12.1%) > Acidobacteriota (11.4%); the relative abundances of the fungi were Mortierellomycota (17.8%) > Basidiomycota (13.4%) > Ascomycota (8.2%) (Figure S1). And the relative abundances of the soil’s bacterial and fungal genera in the different forest types differed significantly (Figure 6, *p* < 0.05). From the bacterial stacking diagram (Figure 6a), *Candidatus* (13.5%) and *Bradyrhizobium* (12.4%) were the dominant genera among all the forest types. Moreover, the relative abundances of *Podila* were ordered as follows: BP > PS > LG > PL. In the fungal stacking diagram (Figure 6b), *Podila* accounted for the highest proportion, which is a fungal genus belonging to the family Mortierellaceae, and it accounted for the highest proportion, especially in the BP; *Peziza* accounted for the highest proportion in the PL, and the remaining three forest types accounted for the highest proportion. *Peziza* had the highest proportion in the PL, and the remaining three forest types had very low proportions.

**Figure 7:**
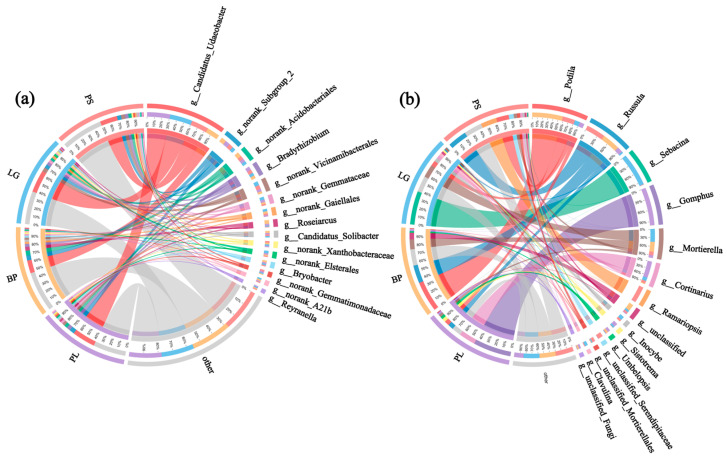
Ring diagrams showing the top 15 genera of soil bacteria (**a**) and soil fungi (**b**) under different treatments. PL: primary pure poplar forest; BP: primary pure birch forest; LG: primary pure larch forest; PS: primary pure sphagnum forest.

The genus ring diagrams of the soil samples in the different forest types (Figure 7) show the percentages of the different microbial species in the various samples: the thicker the line, the greater the percentage of that genus in the sample. As can be seen from Figure 7a, the top three soil bacterial genera with the highest percentages were *Candidatus Udaeobacter*, an unclassified bacterial genus, and a group of archaea. Among the soil’s fungal genera, *Podila* had the highest percentage in all four soil samples, especially in the BP, at 35%; *Russula*, with the second highest abundance, was found in the soil in the PS, at 14.5%; and *Sebacina* had the highest percentage in the LG, at 33% (Figure 7b).

**Figure 8:**
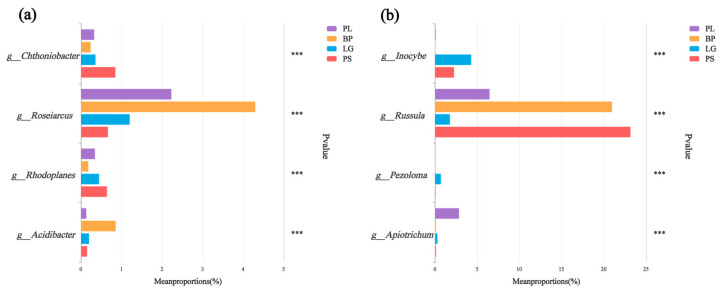
Significant difference test plots between groups were used to test for differences (*p* < 0.05) in the soil’s bacteria (**a**) and fungi (**b**) in the different forest types. Different colors represent different treatment groups. The horizontal axis represents the percentage of genera in different treatment groups, and the vertical axis represents different genus names. PL: primary pure poplar forest; BP: primary pure birch forest; LG: primary pure larch forest; PS: primary pure sphagnum forest. *** *p* < 0.001.

From the test of the intergroup variability, it can be seen that *Chthoniobacter* and *Rhodoplanes* were the highest in the PS; *Roseiarcus* and *Acidibacter* were the highest in BP soils, and *Russula* had the highest percentages in the rest of the soil samples, except for a lower percentage in the LG (Figure 8a). *Inocybe* was the highest in the LG soil and was higher in both conifers than in the PL and BP, and *Pezoloma* had a higher percentage in the PL (Figure 8b).

### 3.4. Relationships Between the Soil’s Microbial Communities and Environmental Factors

**Figure 9:**
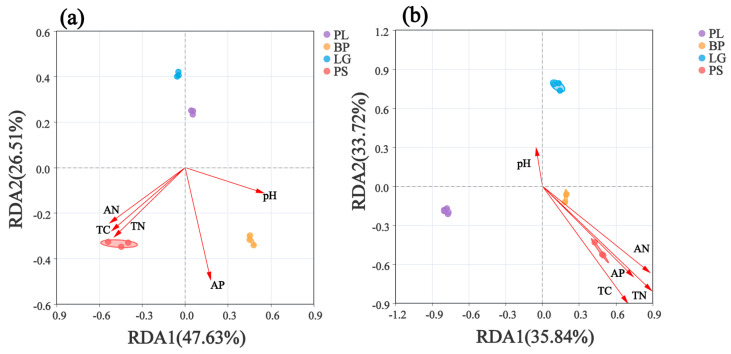
Linear analysis of environmental factor associations for the soil’s bacteria (**a**) and soil’s fungi (**b**). PL: primary pure poplar forest; BP: primary pure birch forest; LG: primary pure larch forest; PS: primary pure camphor pine forest. Each point represents a sample, different color points correspond to different soil samples, and ellipses indicate sample points with 95% confidence intervals. The proximity of two points reflects higher functional similarity between samples. Arrows indicate environmental factors, and the angle between two arrows indicates the magnitude of the correlation between them. An acute angle indicates a positive correlation, a right angle indicates no correlation, and an obtuse angle indicates a negative correlation. The length of the arrows indicates the strength of the effect of the factor on the structure and function of the bacterial community. The angle between the arrow and the axis indicates the degree of the correlation with that axis, with lower angles indicating stronger correlations. The projection of the sample onto the environmental factor arrow reflects the relative value of that factor in the sample. The percentages next to the axes indicate the proportions of the variance in the dataset explained by each axis. TC: total carbon; TN: total nitrogen; AN: alkali-dissolved nitrogen; AP: effective phosphorus.

RDA analysis revealed the relationships between the environmental factors and bacterial and fungal communities in the different forest types’ soils (Figure 9). The soil’s bacterial communities in the BP and PS were positively correlated with TC, TN, AN, AP, and pH, whereas the soil’s bacterial communities in the PL and LG were negatively correlated with TC, TN, AN, AP, and pH (Figure 9a). The soil’s fungal communities in the PL showed a negative correlation with the measured chemical factors. And the soil’s bacterial communities in the BP and PS were positively correlated with TC, TN, AN, AP, and pH and negatively correlated with pH, whereas the soil’s fungal communities in the PL and LG were negatively correlated with pH and negatively correlated with other environmental factors (Figure 9b).

**Figure 10:**
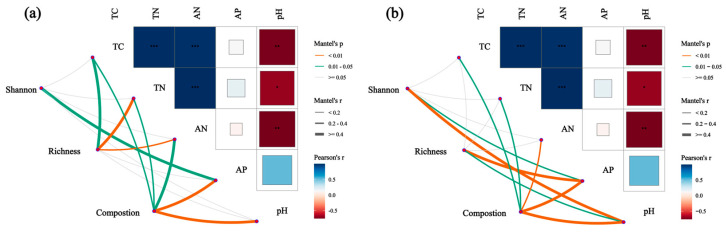
Mantel analysis showing the relationships between the soil’s bacterial (**a**) and soil’s fungal (**b**) community compositions and both α-diversities and the soil’s physicochemical properties. Thicker lines indicate stronger correlations, while thinner lines indicate weaker correlations. PL: primary pure poplar forest; BP: primary pure birch forest; LG: primary pure larch forest; PS: primary pure sphagnum forest. * *p* < 0.05, ** *p* < 0.01, *** *p* < 0.001.

Mantel analysis revealed the relationships between the soil’s bacterial (a) and soil’s fungal (b) community compositions and both the α-diversity and the soil’s chemical properties (Figure 10). Specifically, the bacterial community’s abundance and composition were significantly correlated with TN, AP, and pH. In addition, the bacterial composition was significantly correlated with AP and pH, the fungal community’s richness was significantly correlated with AP, the α-diversity was significantly correlated with pH, and the community’s composition was significantly correlated with AP, pH, and AN.

**Figure 11:**
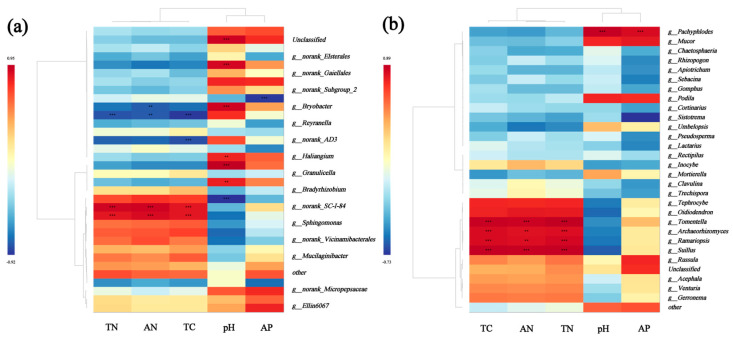
Heatmaps showing the correlations between the top 20 genera of the soil’s bacteria (**a**) and soil’s fungi (**b**) in the soil samples and the soil’s chemical properties. Positive and negative correlations are shown in red and blue, respectively (** *p* < 0.01, *** *p* < 0.001). Correlations were calculated using Pearson’s correlation coefficients. TC: total carbon; TN: total nitrogen; AN: alkali-dissolved nitrogen; AP: effective phosphorus; pH.

Correlation heatmaps show the relationships between the dominant bacterial genera (Figure 11a) and fungal genera (Figure 11b). *Granulicella* and *Bryobacter* of the soil’s bacteria genera were positively correlated with the soil’s pH (*p* < 0.01); *Tomentella*, *Archaeorhizomyces*, *Ramariopsis*, and *Suillus* of the fungal genera were positively correlated with the soil’s TC, AN, and TN; and *Pachyphlodes* was positively correlated with the soil’s pH and AP (*p* < 0.01).

## 4. Discussion

### 4.1. Soil’s Microbial Community Diversity in Different Forest Types

Soil microorganisms, as key players in decomposing organic matter, fixing nitrogen, and improving the soil’s structure, widely influence the balance of biotic and abiotic factors in forest ecosystems [33]. Our results showed that the soil’s bacterial α- and β-diversities changed significantly in the different forest types (Figure 2). Moreover, we found that the α-diversities of the soil’s bacterial and fungal communities were the highest in the PS, followed by the BP, LG, and PL. In the BP, they were significantly higher than those in the PL, and in coniferous soils, the diversity indices in the PS were all significantly higher than those in the LG. This may be because the apoplastic material of birch usually has a lower carbon-to-nitrogen ratio and a lower lignin content than the apoplastic material of other tree types [37], which will provide the soil’s microorganisms with richer AP and more easily utilized carbon sources during rapid decomposition (Table 1). Additionally, this high decomposition rate is conducive for promoting the diversity of the soil’s microbial communities. Ku et al. found that root secretions contain a large amount of organic acids, which also promote phosphorus activation, stimulate mycorrhizal and fungal symbiosis, and indirectly increase both the diversity of the bacterial and fungal symbiosis and bacterial diversity [38]. In contrast, although the decomposition rate of poplar is also higher, poplar secretes phenolic-acid-based chemosensory substances that inhibit the activities of specific bacterial taxa, leading to higher levels of Actinobacteria and lower levels of Acidobacteria [39], and reduce the diversity of the soil’s bacteria in the poplar forest. Similar results were found by Zhu in a study on the soil’s bacteria in four different vegetation communities in the Qilian Mountains [40] and by Liu in a study on the soil’s bacteria in different habitats in the Xiaoxinganling [41]. Nutrients decomposed to apoplastic matter from different plants change the soil’s composition, which, in turn, affects the diversity of the soil’s microbial communities [42]. Organic acids secreted by the root system of camphor pine forests are more abundant, resulting in a lower soil pH than that of larch forests (Table 1), which can rapidly activate insoluble carbon sources in the soil and provide diverse substrates for bacteria [43]. Additionally, the secretions of the larch are dominated by tannins and phenolic compounds; these factors suppress the growth of certain bacteria, resulting in a reduction in the diversity of the rhizosphere soil’s bacterial community.

The trend of the α-diversity of the soil’s fungi was consistent with that of soil’s bacteria, probably due to the fact birch trees have a higher nutrient content and a lower lignin level but a high decomposition efficiency, attracting more saprophytic and ectomycorrhizal fungi to grow [44]. The lower pH of the understory soil in the poplar forest suppresses acid-sensitive flora, such as *Penicillium* spp. [45]. The higher organic carbon content of camphor pine forests provides a rich substrate for saprophytic fungi, such as Ascomycetes and Aspergillus, enabling higher diversity in fungal metabolism. Canini’s research showed that pH is the main factor driving the fungal community [46]. And camphor pine is mainly symbiotic with ectomycorrhizal (ECM) fungi, which mycelial network secretes organic acids, such as oxalic acid, which activate the soil’s effective phosphorus and enhance the efficiency of nutrient cycling [47,48,49]. Larch, on the other hand, relies on tufted mycorrhizal fungi [50], which have a narrower symbiotic range and restricted metabolic activities, and this has led to higher diversity in the PS than in the LG.

The PCoA analysis (Figure 4) showed the variability among the different sample groups. The bacterial compositions of the PL and LG were more similar (Figure 4a), which was consistent with the result obtained by Duan et al. (2023) [51], probably due to the fact that these two species have a common key pathogen (larch–poplar rust), which life history alternates between the two types of trees. Additionally, the sampling time happened to be during the alternating time in order to resist the pathogen attack, which induced the root bacteria of the two species to produce similar resistances accordingly. These two trees belong to the same order genetically [52], so the root secretions also have a high degree of homology, and the response leads to a close distance between the two trees in PCA analysis. The more similar fungal composition of the BP and PS (Figure 4b) may be due to the fact that both rely on exogenous mycorrhizal fungi to construct symbiotic relationships, forming a more similar functional network of fungi. This is also similar to the results obtained by Meidl et al. (2021) [53] and leads to the convergent evolution of fungal communities through the activation of phosphorus in the rhizospherical soil [54,55], which may essentially be because of the synergistic effect of overlapping ecological niches and symbiotic networks.

### 4.2. Soil’s Microbial Community Compositions in Different Forest Types

Our study showed that the content of *Candidatus* was significantly lower in birch forests than in the remaining three forest types (Figure 6a). As a class of warty microfungi, it relies on lignocellulose as a metabolic substrate, whereas birch forests have low lignin contents in their apoplastic matter. Additionally, the competitiveness of *Candidatus* is reduced because of the insufficiency and lower content of the substrate, which is also consistent with the theory of decomposer–substrate interactions proposed by Raczka et al. (2021) [56] and by Wutzler and Reichstein (2008) [57]. The highest content of *Roseiarcus* in the birch forest may be due to the fact that the root secretion of the birch forest is acidic, and the genus *Roseiarcus* shows strong adaptation to acidic soil (Figure 6a and Figure 7a). Boonchuen et al. found that it can utilize the nitrogen metabolism intermediates released by the slow-growing rhizobium genus *Bradyrhizobium* and, at the same time, provide it with a carbon skeleton [58], and this cross-feeding carbon metabolism reduces the resource competition pressure, which explains the survival of *Bradyrhizobium* in birch forests.

Unlike the bacteria’s relative abundance changes, those of the fungi showed obvious forest-type differences. *Peziza*, the most abundant fungal genus in the PL, is more adaptable to the environment, and apoplastic material provides a substrate for spore germination and mycelium customization. Because it often exists as a saprophyte or a weakly symbiotic fungus in the ecosystem, it can form a symbiotic network by mutualistic interaction with poplar roots. *Podila* and *Russula* are widely distributed in various forest types but are predominant in different forest types. *Podila* has the highest relative abundance in the birch forest, which also shows adaptability to the soil’s microenvironment. *Russula* also shows adaptability to the soil’s microenvironment and is widely distributed in the soils of several forest types but has the highest relative abundance in the PS. Li et al. found that *Russula* also plays important roles in the decomposition of organic matter, promotion of nutrient cycling, and maintenance of soil fertility. As a typical example of an ectomycorrhizal fungus that can provide a substrate for mycelia [59,60,61], it is widely involved in carbon and nitrogen cycling and maintaining the stability of forest ecosystems and can significantly increase the effective phosphorus content of the soil by secreting phosphatase to decompose apoplastic material [62], which is also consistent with our test results.

Notably, a large number of unclassified bacteria and fungi were explored in this sampling and testing, which is a common phenomenon in microbial community analysis. To refine our results, we screened for unclassified bacterial genera. The ecological roles of these bacterial groups can be further explored subsequently.

### 4.3. Key Factors Driving Changes in the Soil’s Microbial Communities

Vegetation types directly shape microbial habitats through apoplastic mass and root secretions. For example, broadleaf forests, with low carbon-to-nitrogen ratios and easy decomposition of apoplastic materials, promote bacterial-dominated community development, while coniferous forests, with high lignin contents and slow decomposition of apoplastic materials, are more reliant on fungus-dominated degradation processes, leading to a significant differentiation in microbial compositions between these two types of forests [63,64,65]. Second, the soil’s physicochemical properties, such as the pH, nutrient content, and texture, influence the construction of microbial communities by regulating microbial metabolic activities and resource availability, e.g., the fungal abundance is higher in acidic soils, whereas the bacterial diversity is more prominent in neutral soils. Soil microbial communities are synergistically influenced by multidimensional factors, such as vegetational, environmental, and biological interactions. The bacteria and fungi were all highly correlated with environmental factors (Figure 9), and *Tomentella*, *Archaeorhizomycetes*, *Ramariopsis*, and *Suillus* showed significant positive correlations with TC, AN, and TN. This may be due to the fact that *Tomentella* can accelerate humus decomposition by secreting lignin-degrading enzymes, which play important roles in the soil’s organic matter decomposition and nutrient cycling. Studies have shown that *Archaeorhizomycetes* activates plant root secretions (e.g., malic acid) through symbiotic metabolism to promote the uptake capacity of the soil’s elements by plants and, at the same time, decomposes plant residues in litter into simpler compounds [66,67]. In addition, Wang et al. showed that *Ramariopsis* can form a symbiotic network with mycorrhizae, such as Russula, through synergistic metabolism, which leads to functionally complementary roles and enhances carbon and nitrogen cycling between plants and the soil [68,69].

Mantel analysis (Figure 10) showed that the diversity and abundance of the bacterial communities were mainly influenced by TN and AN, which is consistent with the findings of several previous studies. The soil’s total carbon content showed a significant positive correlation with pH, which was attributed to the fact that pH affects enzymatic activities related to microbial metabolism, which, in turn, affect apoplastic materials’ decomposition. The AP is also an important indicator of the soil bacterial community’s composition. Pang et al. found that soils with higher AP levels exhibited higher fungal diversity, as measured using the Shannon index, and that the AP directly provides key substrates for fungal metabolism, activating both phosphatase and organic acid secretion [70,71]. For example, saprophytic fungi can convert insoluble phosphorus to soluble phosphate by the secretion of citric acid while releasing carbon for other microorganisms to utilize, thus forming a synergistic metabolic network [72]. Soils with high AP levels are usually accompanied by high levels of TN. The decomposition rate is elevated by 25%, while the released ammonium-derived nitrogen further stimulates mycelial expansion [73]. This synergistic carbon–nitrogen–phosphorus cycle provides the basis for the ecological niche differentiation of diverse fungal taxa (e.g., *Ascomycetes*).

Different forest vegetation types provide diverse resource spectra through apoplastic material and root secretions, creating different ecological niches for microorganisms. For example, a broadleaf forest’s apomictic material, with a low carbon-to-nitrogen ratio and easy decomposition, is suitable for fast-growing bacteria to dominate, while a coniferous forest’s apomictic material, with a high lignin content and slow decomposition, is more suitable for microorganisms with a complex degradation ability, such as fungi, to occupy the ecological niche, which leads to a significant differentiation in the microbial communities between these two types of forests [74,75]. Second, the soil’s physicochemical properties (e.g., pH and nutrient content) and climatic conditions (e.g., temperature and humidity) further shape the microbial ecological niche space. In addition, competition, symbiosis, and predatory relationships among microorganisms drive ecological niche differentiation at the microscale, promoting the coexistence and diversity maintenance of microbial communities. For example, Streptomyces inhibits competitors by secreting antibiotics, thereby occupying specific resource niches [76,77], while microorganisms such as Rhizobia expand ecotope ranges through reciprocal symbiosis. In summary, differences in the diversity of the soil’s microbial communities in different forest types are a direct manifestation of ecological niche differentiation, reflecting the multidimensional tradeoffs among microbes in resource utilization, environmental adaptation, and interspecific interactions. This process not only maintains the stability of forests’ soil ecosystems but also provides a functional basis for material cycling and energy flow.

## 5. Conclusions

Our study on the Shuanghe Reserve in the Daxing’anling region of northeastern China revealed how different primary tree species affected the microbial communities of understory soils. As expected, we observed relatively significant changes in the α- and β-diversities of the soil’s microbial communities as a result of different forest types. These changes were accompanied by differences in the relative abundances of specific bacterial genera. For example, the relative abundances of the dominant bacterial phyla varied from site to site, with *Microbacterium wartyi* being the most abundant in the PL soils and the least in the pristine pure birch forests, whereas *Aspergillus* was the most abundant in the BP and the least in the LG soils. The environmental factors affecting the microbial community’s structure differed for each forest type. The bacterial communities of the larch and birch forests were correlated with TN, TC, AN, and pH, whereas their fungal communities were positively correlated with TN, TC, AN, and AP and negatively correlated with pH. This study is the first comprehensive assessment of the soil’s microbial community changes in four types of primary pure forests in the Shuanghe Nature Reserve. Our findings provide important insights into the stability of forest ecosystems in the Daxing’anling region of northern Heilongjiang Province and make a valuable contribution to microbial ecology. It also plays a role in the subsequent exploration of how to protect existing forestry resources.

## Figures and Tables

**Table 1:** Soils’ physicochemical properties for different forest types in the Shuanghe Nature Reserve.

Forest Type	TC (g/kg)	TN (g/kg)	AN (mg/kg)	AP (mg/kg)	pH	C/N
PL	60.10 ± 0.10 b	2.26 ± 0.04 c	243.93 ± 0.47 c	21.15 ± 0.10 c	5.72 ± 0.03 b	27.00
BP	30.04 ± 0.67 d	2.02 ± 0.04 d	184.46 ± 0.42 d	36.36 ± 0.43 a	5.92 ± 0.04 a	15.00
LG	40.92 ± 0.76 c	3.12 ± 0.55 b	354.17 ± 0.18 b	20.09 ± 0.19 d	5.72 ± 0.03 b	15.80
PS	200.54 ± 0.03 a	12.60 ± 0.15 a	896.81 ± 0.42 a	28.06 ± 0.39 b	5.62 ± 0.03 c	16.30

Note: Values are reported as means ± standard errors; different letters represent significant differences between treatments (*p* < 0.05). TC: total carbon; TN: total nitrogen; AN: alkali-dissolved nitrogen; AP: effective phosphorus; pH: Significant differences between experimental groups were determined using ANOVA, followed by pairwise comparisons based on Waller–Duncan post hoc tests. PL: primary pure poplar forest; BP: primary pure birch forest; LG: primary pure larch forest; PS: primary pure sphagnum pine forest.

## Data Availability

The data are contained within the article.

## Supplementary Materials

The following supporting information can be downloaded at: https://www.mdpi.com/article/10.3390/microorganisms13061298/s1, Figure S1: Ring diagrams showing the top 10 phyla of soil bacteria (a) and soil fungi (b) under different treatments. PL: Primary pure poplar forest; BP: Primary pure birch forest; LG: Primary larch pure forest; PS: Primary pure sphagnum forest. Figure S2: The Permanova test of soil bacteria (a) and soil fungi (b) under different treatments. PL: Primary pure poplar forest; BP: Primary pure birch forest; LG: Primary larch pure forest; PS: Primary pure sphagnum forest.
