# Supplementary material for: Variations in the Structure and Composition of Soil Microbial Communities of Different Forests in the Daxing’anling Mountains, Northeastern China
Source: Microorganisms. 2025 Jun 3;13(6):1298. doi: 10.3390/microorganisms13061298 (PMC12195462; doi:10.3390/microorganisms13061298)
Supplement: Supplementary file 1 [file microorganisms-13-01298-s001.zip › microorganisms-3632979-supplementary.pdf]

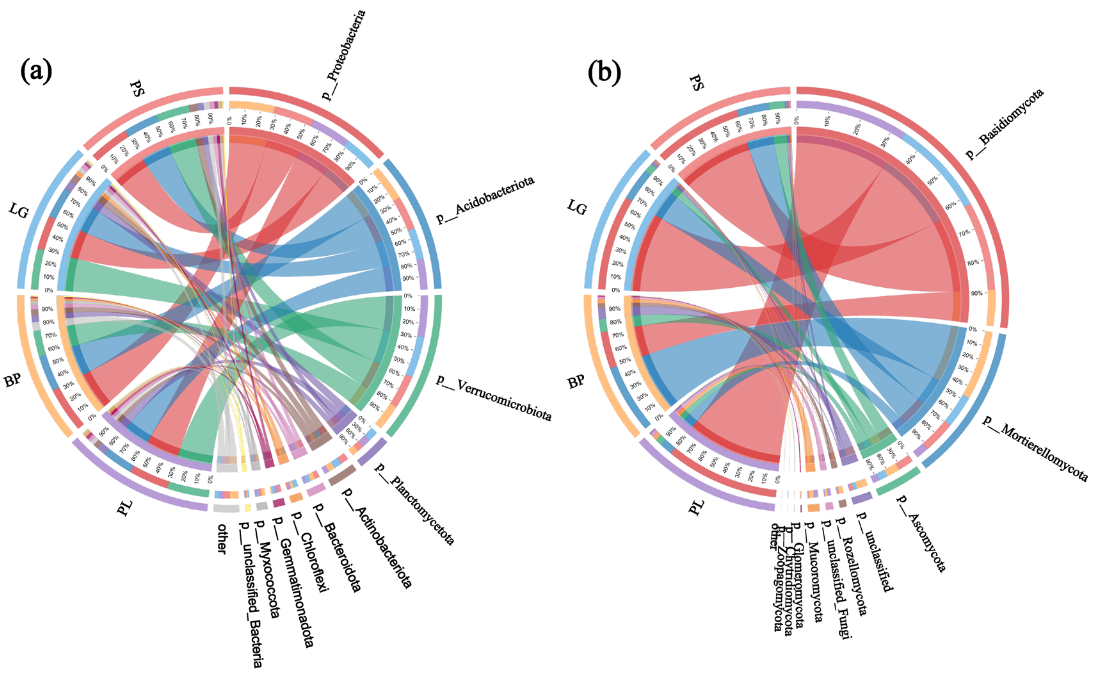

Figure S1 Ring diagrams showing the top 10 phyla of soil bacteria (a) and soil fungi (b) under different treatments.PL: Primary pure poplar forest; BP: Primary pure birch forest; LG: Primary larch pure forest; PS: Primary pure sphagnum forest.

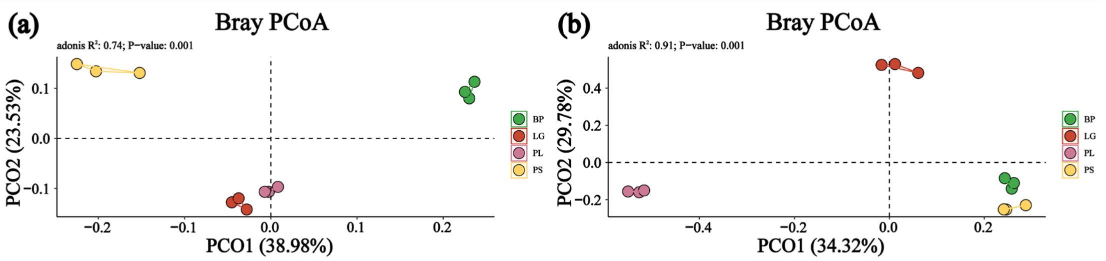

pairs	R^2	p.value	p.adjusted
PL vs BP	0.8426067	0.002	0.0040
PL vs LG	0.7397379	0.003	0.0045
PL vs PS	0.5334734	0.001	0.0040
BP vs LG	0.8446945	0.005	0.0050
BP vs PS	0.6435051	0.002	0.0040
LG vs PS	0.5204877	0.004	0.0048

pairs	R^2	p.value	p.adjusted
PL vs BP	0.9185313	0.001	0.003
PL vs LG	0.9163735	0.004	0.004
PL vs PS	0.8405205	0.003	0.004
BP vs LG	0.9159688	0.003	0.004
BP vs PS	0.8201309	0.004	0.004
LG vs PS	0.8319546	0.001	0.003

Figure S2 The Permanova test of soil bacteria (a) and soil fungi (b) under different treatments. PL: Primary pure poplar forest; BP: Primary pure birch forest; LG: Primary larch pure forest; PS: Primary pure sphagnum forest.
